# Social Information Transmission in Animals: Lessons from Studies of Diffusion

**DOI:** 10.3389/fpsyg.2016.01147

**Published:** 2016-08-04

**Authors:** Julie Duboscq, Valéria Romano, Andrew MacIntosh, Cédric Sueur

**Affiliations:** ^1^Département Ecologie, Physiologie et Ethologie, Centre National de la Recherche ScientifiqueStrasbourg, France; ^2^Institut Pluridisciplinaire Hubert Curien, Université de StrasbourgStrasbourg, France; ^3^Wildlife Research Centre, Kyoto UniversityKyoto, Japan; ^4^Primate Research Institute, Kyoto UniversityInuyama, Japan

**Keywords:** information, sociality, experimental design, social cognition, social network, social competency

## Abstract

The capacity to use information provided by others to guide behavior is a widespread phenomenon in animal societies. A standard paradigm to test if and/or how animals use and transfer social information is through social diffusion experiments, by which researchers observe how information spreads within a group, sometimes by seeding new behavior in the population. In this article, we review the context, methodology and products of such social diffusion experiments. Our major focus is the transmission of information from an individual (or group thereof) to another, and the factors that can enhance or, more interestingly, inhibit it. We therefore also discuss reasons why social transmission sometimes does not occur despite being expected to. We span a full range of mechanisms and processes, from the nature of social information itself and the cognitive abilities of various species, to the idea of social competency and the constraints imposed by the social networks in which animals are embedded. We ultimately aim at a broad reflection on practical and theoretical issues arising when studying how social information spreads within animal groups.

## Introduction to Social Diffusion Theory and Experiments

Many organisms, from plants to social animals, have the capacity to use information provided by others to guide their own behavior or decision (Morand-Ferron et al., 2010). Such information, the behavior of others or its product, constitutes social information. It can be advertently (a signal) or inadvertently (a cue) produced and may complement personal information acquired through trial and error and direct interactions with the environment (Bonnie and Earley, 2007). The use of social information is thought to allow individuals to adapt to their environment faster and/or better than through collecting personal information alone. Use of social information thus provides tremendous evolutionary advantages and is known to occur in many contexts, e.g., regarding food location, availability and palatability, predator threats, and finding and choosing mates (Danchin et al., 2004; Laland, 2004; Dall et al., 2005; Kendal et al., 2005; Bonnie and Earley, 2007; Taborsky and Oliveira, 2012). Even when the information or behavior appears non-adaptive, such as many of the behavioral traditions observed in non-human primates [e.g., hand-clasp grooming (McGrew and Tutin, 1978) or stone-handling (Leca et al., 2012)], such traditions may still be adaptive by preserving group cohesion or reinforcing group membership/identity through conformity for example. In any case, the transmission of such traditions can be under the same social influences as that concerning more obviously adaptive social information. In this review, our main focus is on the transmission pathways of information between one individual (or group thereof) and another, regardless of its ultimate function/adaptive value. However, it must be kept in mind that low adaptive value may in itself partly explain a lack of diffusion of a given behavior, tradition or piece of information, and conversely that high adaptive value may facilitate and even enhance the diffusion process.

Within animal societies, an individual’s ability to use social information and the properties governing its diffusion among group members or conspecifics have been studied under diverse frameworks, from evolutionary psychology (culture, social learning, and communication) and behavioral ecology (public information, eavesdropping) to neuroethology and economics of decision-making (information processing, social influences; Danchin et al., 2004; Dall et al., 2005; Kendal et al., 2005; Bonnie and Earley, 2007; Taborsky and Oliveira, 2012). The common threads binding all of these studies are twofold: (1) the source of information is the behavior of others and (2) the outcome of interest is the change in behavior associated with the acquisition and use of social information (Bonnie and Earley, 2007). Social information is thus a type of biological information, i.e., a property of some source that elicits a change in the state of the receiver in a (usually) functional manner. Differences between fields rest in the information content (who, what, and how) and packaging (signal vs. cue), as well as in the payoffs of using social information (Bonnie and Earley, 2007). For example, an animal’s choice of a feeding site can be influenced by whether or not conspecifics are already feeding there (social influence or social learning), by the conspecifics’ feeding behaviors that may be indicative of resource quality (public information), by how many other animals one can outcompete around the resource (eavesdropping), or by all of the above.

The acquisition and use of social information seems to be inherently adaptive, although some theoretical and empirical examples show that it could also be neutral (e.g., symbolic/arbitrary) and sometimes maladaptive (Rogers, 1988; Giraldeau et al., 2002). A maladaptive decision might also be defined as an inevitable by-products of an adaptive strategy that has evolved under strong selective pressures (Rieucau and Giraldeau, 2011; Pelé and Sueur, 2013). This probably relates to the existence of a trade-off between acquiring costly but accurate information through personal experience and using cheap but potentially less reliable information from others (Barnard and Sibly, 1981; Giraldeau et al., 2002; Laland, 2004; Kendal et al., 2005). Animals must thus adjust the weight they give to both sources of information depending on circumstance. Individuals may rely on social information when personal information is difficult to acquire or unreliable, and when they are uncertain about how to behave. They may instead rely on personally acquired information when the available social information conflicts with it or is incomplete, and/or when individuals are confident in the quality of their own information (Giraldeau et al., 2002; Laland, 2004; Kendal et al., 2005; Rieucau and Giraldeau, 2011). Most likely, decisions involve taking into account a combination of social and personal information and the diffusion of information is thus a function of the cost-benefit ratio of the different strategies available (Rieucau and Giraldeau, 2011). Yellow-bellied marmot (*Marmota flaviventris*) alarm calls, which are given to signal the presence of a predator, provide an opportunity to exemplify this because the caller’s reliability in signaling danger is directly linked to the amount of time others allocate to personally assessing the threat: when the caller is judged unreliable, other marmots spend more time being vigilant (i.e., gathering personal information) before acting (or not) upon the threat (Blumstein et al., 2004). In species establishing recurrent and/or enduring social relationships between group members, reliability of social information also concerns these social relationships. For example, a middle-ranked female rhesus macaque (*Macaca mulatta*) will be more assertive toward an unfamiliar individual if she has seen a familiar subordinate individual defeating it in some competitive interaction (reliable social information), in contrast to conditions in which the interaction involved a familiar dominant or an unfamiliar individual (unreliable social information; cue reliability approach, Dewar, 2003).

Ways of testing functional and mechanistic hypotheses about social information and its use include: observing animals throughout their ontogeny, observing different populations of the same species with different behavioral traditions, or carrying out so-called social diffusion experiments in the lab or in the field. Social diffusion experiments investigate the transmission of social information from one individual (or group) to the next, seeding experimentally controlled innovations in behavior into groups of naïve individuals and tracking and documenting the spread (or otherwise) of the innovation (Whiten and Mesoudi, 2008; Whiten et al., 2016). A traditional experimental paradigm is to have two groups of subjects, an experimental group with a knowledgeable, proficient model that others can observe performing an action, and a control group without such an opportunity to observe. Alternatively, one of several new behaviors is seeded in one or few so-called informed individuals in a group of naïve individuals in order to artificially create behavioral variation amongst groups or populations. The aim is then to track the progressive acquisition of the new behavior in terms of pathways (from whom to whom the behavior is transmitted), speed, accuracy, and characteristics of individuals involved as compared to controls or variants (Whiten and Mesoudi, 2008; Whiten et al., 2016).

In this article, we first review such social diffusion studies and their goals, methods and outputs. We take a broad perspective on such studies, whether observational or experimental, with paired individuals or open groups, in a social learning or public information framework, but try to focus on salient research fitting our aims. We make no attempt to discuss what does or what does not constitute social learning (for comprehensive discussions of this see Galef and Laland, 2005; Hoppitt and Laland, 2008, 2013; Leadbeater, 2015, amongst others), nor to distinguish the mechanisms by which this particular use of social information occurs (see Laland, 2004; Hoppitt and Laland, 2013, amongst others), nor to debate whether the use of social information is adaptive (see Rogers, 1988; Giraldeau et al., 2002; Kendal et al., 2005, amongst others). Hereafter, we instead focus exclusively on the possible pathways for information transmission within groups or aggregations of individuals, and the factors that may enhance or, more interestingly for us, inhibit information transmission. We pay special attention to studies in which the goals and outputs did not necessarily coincide because these studies tell us as much as do studies presenting “positive” results about how animals use, or do not use, social information. In the second part of this article, we return to essential concepts and expand our review on the nature of social information itself, the putative cognitive abilities of various species, the idea of social competency, and the influence of social networks on the use of social information in animal societies (**Table 1**). To paraphrase Bonnie and Earley (2007), our intention here is not to revolutionize the field, but rather to continue stimulating discussions about the abilities of animals to extract, use, and produce information from the social environment, and their influence on information diffusion.

**Table 1:** Summary of points examined in this review.

Transmission process	Known influential factors	Directions for further studies
Initiation	– Producer characteristics (sex, age, dominance rank, and personality, motivation),	– Competing solutions to the same problem
	– Environment (complexity, stability),	– Suboptimal demonstrator characteristics
	– Type of innovation	– Seeding of information to individuals with different characteristics simultaneously
Pathway	– Producer/receiver characteristics	As above, and:
	– Producer/receiver relationships (kinship, dominance difference, “friendship”)	– Several information of varied types (e.g., social/asocial), qualities, relevance, or congruence presented at the same time
	– Cognitive abilities (sensory output and processing)	– Social structure disturbance/manipulation (e.g., alone/in a social setting)
	– Social network (openness, connectedness, tolerance)	– Same type of experiments to many different species/groups (including interspecies)
	– Adaptive value	– Different task complexity/difficulty concurrently
	– Information characteristics	
Establishment/termination	– Cost/benefit ratio,	– Comparison between initial transmission and long-term transmission patterns
	– Conservatism level	
	– Social network structure	

**Additional aspects:**		
– Technological equipment to track non-invasively: individuals’ movements (GPS, accelerometer), physical states (heart rate monitor, blood glucose or glucocorticoid level monitor, infrared imaging), social proximities [radio-frequency identification (RFID) tags]		
– Test apparatus version 2.0 with touch screens or panels, automated feeders, eye-trackers, face recognition		
– Long-term population studies		
– Heritability/evolution/environmental changes studies		
– Taking inspiration in other diffusion domains such as epidemiology, informatics, or social media		
– Building a database of protocols, pre-print, and published studies		


## Social Diffusion Experiments: Goals, Methods, and Outputs

One of the earliest known accounts of social transmission of behavior is milk bottle opening among tits (*Parus major*, *Periparus ater*, and *Cyanistes caeruleus*) in England, where birds learned to pierce the lid of milk bottles left on doorsteps to drink the cream within (Fisher and Hinde, 1949; Aplin et al., 2013). Although this innovative behavior started in several places independently, once present in a population it would spread extensively, suggesting the influence of social processes (Fisher and Hinde, 1949; Lefebvre, 1995; Aplin et al., 2013). Another known example of social transmission among animals comes from Japanese macaques (*Macaca fuscata*) washing sweet potatoes in water, a behavior that spread gradually through the group (Kawai, 1965). In the years following the start of this seminal study, several other newly acquired behaviors (e.g., begging, stone-handling) emerged and spread through different groups of macaques in different regions of Japan following rules of acquisition dependent mainly on age, sex, and kinship (Kawai, 1965; Huffman et al., 2008). Since then, almost all published experimental or natural studies of social information transmission show that given the possibility to observe knowledgeable individuals performing a task, the majority of naïve, non-knowledgeable individuals subsequently use the same technique to accomplish the same task (Morand-Ferron et al., 2010). The non-random process of task acquisition is generally demonstrated if it occurs either above chance or above the proportion of naïve individuals performing the same task in a control group without knowledgeable demonstrators or in a group seeded with a different technique (Whiten and Mesoudi, 2008; Whiten et al., 2016). These results seem to be taxon-independent and pertain to insects, birds and mammals, demonstrating the overwhelming generality of social information use by animals (Laland, 2004; Chittka and Leadbeater, 2005; Galef and Laland, 2005; Whiten and Mesoudi, 2008; Rieucau and Giraldeau, 2011; Whiten et al., 2016). We can nevertheless distinguish these studies into three, non-exclusive categories: (1) those relating to the presence/absence of diffusion of the behavior; (2) those regarding individual characteristics and their influence on transmission; and (3) those interested in the pathways and characteristics of diffusion (e.g., persistence of transmission). Complementary to the ideas presented here, Whiten and Mesoudi (2008) and then Whiten et al. (2016) also provide extensive and updated reviews of diffusion studies in animals and humans.

### Presence/Absence of Diffusion

A first step in studies of social diffusion is to show that information is actually transferred amongst animals in some way. The literature is vast and spans contexts such as foraging, breeding, anti-predation strategies, and social interactions. Examples range from bumblebees (*Bombus impatiens*) choosing the same-colored flowers as those chosen by conspecifics they previously observed (e.g., Leadbeater and Chittka, 2005; Worden and Papaj, 2005), to client fish (*Scolopsis bilineatus*) spending more time near cooperative cleaner fish (*Labroides dimidiatus*) than cleaner fish of unknown cooperative level after observing other clients’ interactions with these cleaner fish (e.g., Bshary and Grutter, 2006), to flycatchers (*Ficedula albicollis*) using others’ breeding outcomes (offspring quantity and/or quality) to select a breeding habitat (e.g., Doligez et al., 2002).

The interest here lies in where transmission apparently did not occur, because looking at how, why, and in what context animals do not use social information is just as telling as when they do. For instance, wild keas (*Nestor notabilis*), a mountain parrot, failed to solve a foraging task despite having the opportunity to observe proficient individuals solving the same task and to engage with the experimental setup immediately thereafter (Gajdon et al., 2004). When the experiment was repeated with captive keas, a majority of the birds solved the task after observing a proficient model (Huber et al., 2001; Gajdon et al., 2004). This indicates that the absence of social information transmission was independent of the task’s level of difficulty. It could be that wild keas have the capacity to learn socially but some constraints prevent them to express it – maybe a question of opportunity or utility. This is similar to what is found in spotted hyenas (*Crocuta crocuta*), a social carnivore, where individuals in captivity seem more proficient at solving foraging tasks than those in the wild. This difference was attributed to personality rather than more trivial factors such as time-energy threshold, inasmuch as captive hyenas are more exploratory and less neophobic than their wild counterparts (Benson-Amram et al., 2013). In contrast, a novel foraging behavior (piercing a lid to access food) spread more quickly amongst groups of free ranging urban pigeons (*Columba livia*) than amongst captive groups. This was explained by the fact that urban pigeon groups are open to migrants which could enhance the degree of innovation and diffusion (Lefebvre, 1986).

Looking in more details at the hyena example, whether in captivity or in the wild, individuals presented with a box containing meat were more likely to approach and manipulate the box when they had seen others do it but were not more likely to succeed in opening it (Benson-Amram et al., 2014). In this case, social information is used indirectly to enhance extraction of personal information but not directly to solve an environmental problem. This could be explained by the simplicity of the task (solvable by trial and error), or the characteristics of the demonstrator (not relevant or reliable). It could also be that social constraints, such as a rather competitive environment, affects the cost/benefit ratio of social information vs. personal information: hyenas are very good at solving goal-oriented cooperative tasks (Drea and Carter, 2009), which may be necessary to hunt large prey, but when they already have access to food, they may instead pay more attention to avoiding aggression than to new ways of obtaining the food *per se*. A lack of diffusion and establishment of a behavioral pattern can also occur when two alternatives are equally profitable. In meerkats (*Suricata suricatta*), individuals were at first more likely to feed on the same feeder as a demonstrator, but the more they explored the experimental apparatus, the more they realized they could easily get food at two “locations,” making it less likely they would continue to use the demonstrator’s feeder more frequently (Thornton and Malapert, 2009). In this example, although there was social transmission from one demonstrator to one observer, there was no establishment of behavioral tradition such that the behavior spread within the whole group according to individual’s assortativity.

In other cases, the task presented seems too difficult, not appropriate or not ecologically relevant for the tested animals. For instance, laboratory-reared rhesus monkeys learned to fear snakes from watching videos of wild-reared conspecifics’ reactions to snakes, but never learned to fear a flower on the same basis (Cook and Mineka, 1989). In a two-step foraging task, vervet monkeys (*Cercocebus aethiops*) had to remove a rope blocking a door before opening that door to retrieve food. Although the trained model was ultimately successful at the task, other individuals failed to master it although they were exposed to a successful model, suggesting that the link between one gesture and the next in a several-steps task was not evident (van de Waal and Bshary, 2011). Another example of a behavior, this time naturally occurring, that failed to spread is dental flossing in Japanese macaques (Leca et al., 2010). In their study, the authors reported several factors likely to constrain the diffusion of innovation such as belonging to a small grooming cluster relative to group size or having few close kin in the group, and the form, function and context of the behavior. The most interesting point that the authors made here is that the low adaptive value of dental flossing, a “comfort” innovation with such a “narrow window of applicability,” may also account for its lack of diffusion (Leca et al., 2010).

### Influence of Individual Characteristics on Diffusion

Because social groups are often mixed groups of individuals of different sexes, ages, and/or personalities, individuals’ interest in, and experience and knowledge of, their environments vary. Thus, some individuals are potentially more likely to discover resources in the environment, to start innovating, or to correctly assess dangers than others, creating a differentiation in the availability and reliability of the social information produced within a group/aggregation of animals. On the other hand, some individuals are also more likely to learn from their conspecifics because they are more social (in general terms), i.e., they are more often in proximity to others, they pay more attention to others, or they are more often engaged in social activities.

For instance, only 54% of naïve blue tits exposed to a proficient demonstrator solved a new foraging task (Aplin et al., 2013). Investigation of the variables that could explain this percentage showed that young females and subordinate males with higher innovative problem-solving capabilities were more likely to solve the task than others, whereas the characteristics of the demonstrator had no influence on the performance of naïve birds, i.e., there was no preferential attention to certain models (Aplin et al., 2013). On the other hand, studies on vervet monkeys demonstrated that social transmission is often influenced by kin relationships, i.e., vertical, from mother to offspring (van de Waal et al., 2014). When transmission is horizontal, from peer to peer, or oblique, from adults other than parents, vervet monkeys are more likely to copy the new foraging technique of an adult female compared to an adult/subadult male (van de Waal et al., 2010). Adult females of this species are philopatric and live their entire lives in the group in which they were born. This potentially makes them more reputable concerning food acquisition and processing because they have more experience and are the more familiar individuals in the group. They could also occupy more central positions in the social network of the group and may be more tolerant of individuals in proximity, all of which could potentially enhance social information transmission.

Similarly, it has been experimentally shown that, visually, monkeys do attend more to higher-ranking individuals than to lower-ranking individuals (e.g., McNelis and Boatright-Horowitz, 1998; Deaner et al., 2005), and to strong affiliates compared to average affiliates (Bonnie and de Waal, 2006; Micheletta et al., 2012). This pattern is interpreted as being more salient in terms of acquiring social information. As another case in point, the oldest living female in a group of African elephants (*Loxodonta africana*), the matriarch, often leads the group from one place to another and initiates group defense behavior (for example when encountering signs of unfamiliar individuals or of predators), potentially because she has enhanced local knowledge of the environment and group members defer the decision of travel/action to this informed individual (McComb et al., 2001, 2011; Mutinda et al., 2011). However, the best innovators, i.e., individuals more likely to start using a novel behavior, are not necessarily the best models for information transmission. For example, although male canaries (*Serinus canaria*) were better at solving a foraging task and thus could have been selected as demonstrators, their aggressive tendencies toward others prevented them from being good models (Cadieu et al., 2010). In this case, females constituted the best demonstrators because they tolerated individuals around them, so social transmission of an innovation mainly rested on females.

### Diffusion Pathways

When social information is transmitted, determining the pathways taken by this information within a group of individuals as well as how fast and far it travels can give insights into the mechanisms of social information use. Indeed, animals living in groups or aggregations do not interact or associate randomly with one another, but have preferred associates or affiliates which are reflected in the heterogeneous structure of the social network of the group/aggregation. As such, the flow of social information is not random between individuals, but is in accordance with the structure of the social network of the population (Krause and Ruxton, 2002; Krause et al., 2007; Croft et al., 2008). Social transmission of information can thus fail not only because of some characteristics of demonstrators and/or naïve individuals, but also because the link between knowledgeable and naïve individuals may be suboptimal, e.g., the pair is not often together, not strongly affiliated or even avoids association, whatever the underlying causes. “Where” [i.e., with which individual(s)] to seed the social information diffusion within a network of individuals is thus as crucial as how connected the individuals are.

In brown capuchins (now *Sapajus apella*) for example, transmission during diffusion chain experiments was controlled in that pairs of demonstrators-observers were chosen amongst affiliates and the demonstrator was the higher-ranking of the two, which may have facilitated transmission (Dindo et al., 2008). In contrast, in a group of squirrel monkeys (*Saimiri sciureus*), where the chosen demonstrator of a new foraging technique was the alpha male, the open diffusion experiment demonstrated that more central individuals in the social network (those well connected and integrated in the group) were more successful at mastering the technique and quicker at using it than less central individuals (Claidière et al., 2013). Central individuals indeed may have more opportunity to observe the demonstrator and/or to manipulate the apparatus, especially if the demonstrator is itself central, which would enhance the use of social information. In a more natural setting, Brown (1986) showed that cliff swallows (*Hirundo pyrrhonota*) that were unsuccessful at bringing food back to the nest for nestlings were more likely to follow a successful individual on their next foraging trip than were successful foragers. Unsuccessful foragers were also more likely to follow their nest neighbors on subsequent trips, especially those within 1 to 5 nests away than further away in the colony. As there was intra-individual variation in foraging success, any bird could be a successful or unsuccessful forager and thus a follower or a leader to a foraging patch. This led Brown to coin the swallow colonies as “information centers” and is one of the earliest examples of diffusion analysis in a foraging context, albeit in a crude way (Brown, 1986).

A major step forward in the study of social diffusion is the development of network-based diffusion analysis (NBDA). NBDA is a tool now commonly used to demonstrate that the expression of a behavior by an individual is the result of it being associated with animals that themselves express this behavior with an increased probability compared to a model not including social effects (Franz and Nunn, 2009; Hoppitt et al., 2010). The model specifically illustrates directed social learning, in which information is transmitted at different rates depending on association patterns between individuals (Coussi-Korbel and Fragaszy, 1995). Such social effects explain variance in lobtail feeding in whales (Allen et al., 2013) or food patch discovery in tits (Aplin et al., 2012). The latter study not only demonstrated that tits use social information to locate new food patches but also that the discovery success was linked to individual centrality in the flock association network: more central individuals were more likely to locate and use novel foraging patches than those with limited social connections. By looking at an animal or human group as a network of connected individuals, social network analysis has facilitated great progress in diffusion studies, and as a result, in the understanding of animal and human culture. Because culture is fundamentally based on the exchange of social information, social structure and culture are indeed linked (Cantor and Whitehead, 2013). In this perspective, diffusion studies, whether experimental or observational, coupled with social-network-based analysis brought substantial advances to our understanding of how animals use social information.

## Further Perspectives on Social Diffusion Studies

Questions regarding the acquisition and use of social information are typically concerned with when to copy (e.g., when resources are easy or difficult to exploit/find, or when the environment is stable or unstable), who to copy (e.g., successful or reputable or familiar or genetically related individuals), what is copied (i.e., what kind of information is remembered and transmitted) and how individuals copy (i.e., the mechanisms or supports by which the information is reproduced; Laland, 2004; Bonnie and Earley, 2007; Whiten and Mesoudi, 2008; Whiten et al., 2016). The literature covering each of these aspects is vast and continues to expand almost exponentially (Galef, 2012; Whiten et al., 2016). The challenge that remains even today is to examine those questions in more integrative ways and to find the right experimental, empirical, and statistical paradigm to do so (Whiten et al., 2016).

Important aspects of diffusion that we feel deserve more attention include social information characteristics, what makes an animal a producer and/or a user of information, the cognitive capacities involved in acquiring, processing, and using social information, and finally the social competency of animals. We also think that future work could pay more attention to quantifying the rate at which information spreads, how far this information can spread in a network, and the factors that influence the flow of information. This means that an additional focus to factors favoring social transmission could be on those explaining an absence thereof. We now turn to these topics in a humble attempt to participate in advancing the field of social information use in animal societies.

### Social Information Characteristics

The characteristics of social cues, i.e., information that is inadvertently produced through interaction with the environment, can greatly influence their transmission inasmuch as acquiring and using social information is directly related to the cost of acquiring and using asocial or personal information (Boyd and Richerson, 1988). These characteristics can be experimentally modified to assess which are important to the animals. For example, is the number of conspecifics performing a task sufficient, or are subtler cues necessary to decide to use social information? For instance, experiments of social transmission in fruit flies (*Drosophila megalonaster*) showed that within an aggregation, the number of informed individuals needed to be about twice the number of uninformed individuals in order to observe transmission of information from informed to uninformed individuals (Battesti et al., 2015). Experiments with fish and birds demonstrate that individuals without *a priori* information on environmental resources are more likely to follow a large group of conspecifics to a food location compared to a small group. But as soon as individuals can observe others actually feeding, they would rather follow few individuals feeding than many individuals not feeding (Kendal, 2004; Coolen et al., 2005; Rieucau and Giraldeau, 2011). This suggests that observing a direct link between a task and a reward is more salient than just observing a task. Similarly, individuals with *a priori* personal (or asocial) information are less influenced by their companions’ behavior than those without. In an experiment with nutmeg mannikins (*Lonchura punctulata*), individuals without prior personal information consistently chose the feeder associated with previously acquired social information regardless of whether it was the mere numbers of companions present or the numbers of companions feeding. Individuals with prior personal information, however, did stick to their initial choice and switch feeders only if they observed companions actually feeding (Rieucau and Giraldeau, 2009). More subtly, homing pigeons were shown to adjust their flight routes, to which they generally show high fidelity, depending on those followed by conspecifics (Biro et al., 2006). When the pre-established routes of two pigeons did not differ greatly, a pair would converge on an average path, supporting the “many-wrong” hypothesis arising from a compromise between personal and social information. However, as soon as the routes diverged beyond a distance threshold, one individual became the leader, usually the pigeon most faithful to its own pre-established route, supporting the leadership hypothesis in which the most insistent, “confident,” or less flexible individual imposes a social choice on the group. In other cases, both pigeons defaulted to their established routes and thus no use of social information was observed, again usually when the routes diverged beyond a distance threshold (Biro et al., 2006; Freeman et al., 2011).

Another characteristic of information that is likely to influence its transmission is complexity or difficulty. A one-step task may thus be acquired and spread faster between individuals than a task requiring four steps to be completed. For example, callitrichid monkeys used social information to solve a challenging foraging task involving pulling a door toward oneself and retrieving food inside a box, whereas they solved an easier foraging task involving pushing a door and reaching inside to retrieve food without using social information (Kendal et al., 2009). Similarly, vervet monkeys easily solved a simple foraging task such as pushing/pulling a door (van de Waal et al., 2013), but failed to solve a two-action foraging task, even when being provided with social information (van de Waal and Bshary, 2011). Information complexity or stability can also emerge from the environment. For example, the structure of the environment (open vs. closed, arboreal vs. terrestrial) can influence how communication signals can be perceived (Maciej et al., 2011). Starlings (*Sturnus vulgaris*) in an unpredictable environment are better at foraging when in the presence of an informative demonstrator (who consistently indicated the same food location) than in the presence of an uninformative demonstrator, whereas individuals in a predictable environment performed equally well with or without an informative demonstrator (Rafacz and Templeton, 2003). The extent to which the complexity or stability of the environment affects the transmission speed, accuracy, and reach of social information is still not very clear, however. Ecological and social environments may very well interact to affect social information transmission inasmuch as an individual’s perception and action are tightly linked to both (e.g., Barrett, 2011).

Some types of information are also more salient or relevant than others, which will influence their social transmission. For example, humans recall and repeat social information such as gossip involving third-parties with greater accuracy and in greater quantity than non-social information such as the geographical description of a city (e.g., Bartlett, 1932; Mesoudi et al., 2006). In animals, several studies hint that individuals would probably also pay more attention to information relating to social events as opposed to non-social events. For example, fish choose to take a long circuitous route with their mates rather than a shorter more direct route alone to access food. This preference persists over several generations even when founder demonstrators have disappeared from the population (Laland and Williams, 1998). Similarly, in a two-choice test paradigm where male rhesus macaques had to choose between receiving a fruit juice reward or receiving a fruit juice reward and seeing an image of a conspecific, they not only chose the latter option but sacrificed a bit of the amount of juice they could have received to do so (Deaner et al., 2005). This choice demonstrated that monkeys were ready to sacrifice a food reward to gather social information.

Another characteristic we briefly mentioned before concerns the adaptive value of a given piece of social information. If social information that is obviously adaptive, e.g., use of a tool to extract food among primates and corvids, versus that which is not-so-obviously adaptive, e.g., stone-handling among Japanese macaques, were to be seeded in the same group or aggregation, would the spread, speed and reach of diffusion of the former be more important than the latter? The relevance of the former compared to the latter would intuitively lead us to predict a positive relationship between adaptive value and these diffusion properties. However, if these not-so-obviously adaptive socially transmitted behaviors play a role in increasing group cohesion through conformity for example, the answer may not be so straightforward.

So, in general, although animals can display great interest in an experimental apparatus or a given situation, perform a task or a behavior to perfection, and readily observe and copy others, we still know too little about the nature of social information and its influence on transmission dynamics to predict when these behavioral aspects will coincide and result in diffusion. Is it about quality, quantity, complexity, congruence, relevance, or a mixture of all of these traits? Determining this requires long and patient trial-and-error tests, massive undertakings of experiments encompassing varied conditions, contexts, and characteristics, mathematical models and efforts in complex systems science and, importantly, although the information can sometimes be extracted from the study itself, a systematic report or test of the kind of information that is tested/used. Experiments combining tests of asocial and social information simultaneously are also important in determining characteristics of diffusion as it is likely that animals use a combination of both at every instant (Rieucau and Giraldeau, 2011).

### Animals as Information Processors and Users

Each step of the transmission process requires individuals to “innovate” on a personal level, that is, they are not necessarily the first to express the behavior but this is the first time that they themselves express it. In this sense, understanding limits to innovation helps understanding constrains on social diffusion (Brosnan and Hopper, 2014). One of these limits is within the animals themselves, related either to individual characteristics – explored in this section – or to cognitive abilities – explored in the next section (for limits concerning the social environment, see “The social competency of animals or the social network effect”).

Characteristics of the information producers, such as relative status, age, or sex, cannot only influence the performance of an individual in its environment but can also condition another animal’s decision to observe such producers and to use the information gathered. Similarly, characteristics of the information receiver determine its processing and use of information and, as such, the speed, accuracy and extent of information transmission. Individual constraints on social diffusion (here, of innovations) stem from the propensity of individuals to be conservative, that is, individuals tend to persist with existing behaviors, or the existing uses of behaviors, rather than explore novel options (Brosnan and Hopper, 2014). As a case in point, bolder and less neophobic individuals are more likely to produce information and to innovate than shy and neophobic individuals because they tend to take more risks and explore their environments more (Wilson, 1998). Lower-ranking chimpanzees tend to be more innovative, probably because they are more constrained in their access to food and have to find an alternative solution more often than higher-ranking individuals (Reader and Laland, 2001). In great tits, variation in spontaneous problem-solving performance was unrelated to individual state (e.g., body condition) and not even associated with behavioral traits (e.g., neophobia), but most likely reflected inherent individual differences in the propensity to forage innovatively (Cole et al., 2011). In starlings, less neophobic and higher-ranking individuals were more likely to approach the experimental novel foraging tasks. Group mates of these first “contactors” approached the experimental apparatus more quickly as well if they themselves had a propensity to feed in a novel environment (Boogert et al., 2008).

Nevertheless, although some studies have determined which individuals tend to learn or innovate faster or better (see references in previous paragraph), we are still at risk of making a lot of assumptions about who those individuals might be instead of testing who they actually are. When studying animals living in group, especially in natural conditions, researchers are indeed often constrained in the choice of knowledgeable demonstrator(s) vs. naïve observer(s), because high-ranking individuals monopolize the resources for example, or because bolder individuals are more explorative. It is also very difficult to disentangle which individual characteristics can have the most influence, as high-ranking individuals for instance can also be bolder than low-ranking individuals. Studies conducted with wild animals must keep these sociodemographic constraints in mind when being discussed or reported. Finding ecological validity in diffusion studies is a much needed challenge (Whiten et al., 2016).

Overall, what makes a producer and/or a user of information varies greatly according to ecological, social, and individual circumstances. What we need to be more aware of is that not all individuals will produce or use social information, in relative and absolute terms. Optimizing our knowledge and understanding of the speed, accuracy, and spread of social information transmission requires that the profiles of producers and users be more systematically reported. We also need studies that can select producers and users with suboptimal characteristics, for example a high-ranking individual with a lower-than-expected network centrality compared to a low-ranking individual with a higher-than-expected network centrality, or a lower-ranking individual with a higher-than-expected boldness profile compared to a high-ranking individual with a lower-than-expected boldness profile. For instance, in several groups of vervet monkeys tested in an experimentally induced coordination problem, dominant individuals naïve to a foraging task learnt to wait outside of an imaginary forbidden circle that the proficient but low-ranking individual approached and solved the task and allowed food access to the whole group (Fruteau et al., 2013). What is also needed is the assessment of the effects of individual characteristics on diffusion in naturally or spontaneously occurring innovations, observed from their birth to their establishment or disappearance, in a population where individuals are identifiable and their characteristics *a priori* known [e.g., dental flossing (Leca et al., 2010) and louse egg-removal techniques (Tanaka, 1998) in Japanese macaques, lobtail feeding in humpback whales (Weinrich et al., 1992; Allen et al., 2013), or moss-sponging in chimpanzees (Hobaiter et al., 2014)].

### Cognitive Abilities

The social brain hypothesis states that increasing social complexity drives the evolution of large brains with more cognitive capacities, in the sense of information-processing, because of the challenges of managing complex social relationships (Whiten and Byrne, 1997; Dunbar, 1998; Pérez-Barbería et al., 2007). However, the use of social information is so widespread in the animal kingdom that one could contend that information-processing capabilities do not relate only to brain size (Barton, 2006; Morand-Ferron et al., 2010; Lihoreau et al., 2012). The fact that invertebrates such as wasps and bees are capable of memory and learning demonstrates how complex cognitive processes are possible even with a limited number of neurons (Lihoreau et al., 2012; Avarguès-Weber and Giurfa, 2013; Grüter and Leadbeater, 2014). Paper wasps (*Polistes fuscatus*) can recognize individuals and remember the identity of social partners, even after a succession of interactions with other individuals (Sheehan and Tibbetts, 2008). Honey bees (*Apis mellifera*) are well known for their symbolic “dance language,” which they use to build consensus about relocating to a new home: the swarm integrates the different information given by different explorative scouts through their dancing and make a decision about a single location (Seeley, 2010). In the field of social learning, it has been argued that social learning does not depend on “advanced” cognitive adaptations, and that social and asocial learning alike depend on the same mechanisms (Heyes, 2012). This hypothesis is supported by the facts that social and asocial learning abilities covary across and within species (Bouchard et al., 2007; Reader et al., 2011), that social learning occurs also in solitary animals (Fiorito and Scotto, 1992; Wilkinson et al., 2010), and that social learning has the same key features in diverse species, including humans (Heyes, 1994, 2012). Heyes (1994, 2012) therefore argues that social and asocial learning depend on a common set of associative learning mechanisms and that social learning merely reflects the case in which the information is provided through a social channel (Heyes, 2012). This illustrates how the use of social information may in fact require relatively simple and computationally inexpensive forms of cognition (Lihoreau et al., 2012).

However, the use of social information also involves perceptual, attentional, and motivational processes specific to information coming from other individuals (Heyes, 2012). Acquiring and using social information requires animals to link other individuals’ actions to environmental and/or social reactions or patterns. Feedback from the social domain also requires that individuals integrate and process stimuli not only related to the external (e.g., sex, size) but also to the internal (e.g., “emotional”) states of other interacting agents, to the current social context, and to what this information means to the individual at that moment in time in order to respond with the appropriate behavior (Trimmer et al., 2008; Clutton-Brock, 2009; Taborsky and Oliveira, 2012). Throughout the evolutionary history of social species, these social-specific processes may have been selected for and may have further coevolved with the complexity of social life (Heyes, 2012; Leadbeater, 2015). For instance, Pinyon jays (*Gymnorhinus cyanocephalus*), a social corvid species, perform a social learning task better than an asocial learning task whereas Clark’s nutcrackers (*Nucifraga columbiana*), a less social corvid, perform equally well in both tasks (Templeton et al., 1999). Based on these differences, social learning capabilities were interpreted as being adaptations to social life (Templeton et al., 1999; Heyes, 2012). This is essentially one of the tenants of the cultural intelligence hypothesis (Whiten and van Schaik, 2007; van Schaik and Burkart, 2011), which examines links between asocial and social learning and the development and maintenance of learned skills both horizontally and longitudinally in an effort to better understand the emergence and maintenance of cultures and traditions.

From a neuroethological perspective, some parts of the brain are specifically dedicated to social stimuli, such as face recognition and processing, social approval (i.e., individuals tend to conform to social norms to “fit in”), selective social attention (e.g., individuals pay more attention to higher-ranking individuals), or recognizing and responding to socio-emotional signals such as fear and anger (Brothers, 1999; Insel and Fernald, 2004; Phelps and LeDoux, 2005; Barton, 2006; Adolphs, 2008; Rilling and Sanfey, 2011). Mirror neurons are specifically activated both when one performs an action such as reaching for food and when one observes someone else performing that same action (Gallese, 2007; Caggiano et al., 2009). In the broadest sense, emotions are “an evaluative response of the organism involving physiological arousal and expressive behavior,” and “interfacing between sensory inputs and motor outputs in a way that allows flexibility in the response (to a stimulus)” (Aureli and Schino, 2004 for one definition amongst many). They function as adaptive responses to environmental demands, preparing individuals to cope with challenges (Aureli and Whiten, 2003; Aureli and Schino, 2004; Phelps and LeDoux, 2005; Naqvi et al., 2006; van den Bos et al., 2013). As shown in many (natural or induced) experiments of brain lesions/malfunctions in humans and animals (e.g., in the case of autism or brain damage due to an accident), individuals that are physiologically or neurologically stressed or impaired have difficulties making decisions in the social domain and may thus be poor users of social information, which would ultimately constrain social information diffusion without giving any indication about their cognitive abilities. For example, individuals with a damaged ventromedial prefrontal cortex have normal intellect and problem-solving abilities under test conditions in the lab, but make unfortunate decisions in real-life situations and do not learn from their mistakes. This is due to the fact that they have a generally “flat affect” and are thus unable to use emotions to aid in decision-making (Damasio, 1994; Naqvi et al., 2006).

From these perspectives, focusing social cognition research on sensory information, computational challenges, and neural networks, i.e., brain functioning, would be a rewarding way of looking at animal cognitive abilities in the social domain (Chittka and Niven, 2009; Barrett, 2011; Lihoreau et al., 2012). Designing experiments and observations where animals’ motivational, emotional and perceptual capabilities concerning their social worlds are accounted for could give important insights into how social information is transferred within a group.

### The Social Competency of Animals or the Social Network Effect

Ingenious mathematical models and experimental designs show that efficient transfer of information and decision-making can occur within animal groups in the absence of individual recognition, advanced cognitive abilities or complex mechanisms of transfer, and that individuals can respond spontaneously to others that possess information. All that is needed is variation in information holding among members of a population and simple mechanisms of coordination (e.g., Couzin et al., 2005).

However, these kinds of simple decision rules are more likely to be present in societies where individuals do not form differentiated relationships with each other. When group members have the opportunities to recognize each other and memorize past interactions that influence future ones, they do form differentiated relationships that can condition and influence their decision-making processes (Sueur, 2011; Lee and Harris, 2013; Pasquaretta et al., 2014). The heterogeneous distribution of social connections within a group also creates heterogeneous opportunities to observe and learn from certain individuals (as in directed social learning, Coussi-Korbel and Fragaszy, 1995). As such, the structure of the social network of a group can have important consequences for the social transmission of information (Coussi-Korbel and Fragaszy, 1995; Croft et al., 2008; Aplin et al., 2013; Cantor and Whitehead, 2013). For example, observer deer mice (*Peromyscus maniculatus*) have stronger reactions of preparatory analgesia and self-burying in reaction to biting flies when the observer is genetically related to or is more familiar with the demonstrator, although the demonstrator’s behavior does not vary with social conditions (Kavaliers et al., 2005). High-ranking rhesus macaques solve a color-discrimination problem equally well when in a whole group or only amongst high-ranking individuals, whereas low-ranking individuals perform better when with other low-ranking individuals only than when with the whole group (Drea and Wallen, 1999). In a cooperation task, spotted hyenas adjust their behavior to the skills and capabilities of their partners (for example, when an adult is paired with a youngster) and their level of cooperation is modulated by the composition of their social group inasmuch as an individual’s performance is better predicted by the presence of high-ranking individuals – which can be quite aggressive – than by the subject’s prior experience in the task to solve (Drea and Carter, 2009). An entire field of research in animal communication is dedicated to these moderating effects of social context, so-called “audience effects,” i.e., individuals adjust their decisions or behaviors depending on who is with or around them (Zuberbühler, 2008). Conformity, i.e., doing what the majority does, is a very influential mechanism by which culture emerges, evolves and persists (Laland, 2004; Morgan and Laland, 2012). Reaching a consensus decision, on where to go for example, is also a well-studied example of social modulation of behavior (Conradt and Roper, 2009).

Social network analysis (SNA) has proven a useful and powerful tool in understanding social influences on the patterns of acquisition and use of social information (Croft et al., 2008; Voelkl and Noë, 2010; Kurvers et al., 2014; Brent, 2015). A simulation study based on a substantial dataset of primate interaction matrices tested the hypothesis that the social structure of a group has a strong influence on patterns of social learning (Coussi-Korbel and Fragaszy, 1995) by comparing information flow within networks in empirical (structured) social groups and theoretical well-mixed groups in terms of propagation speed, path length of transmission and resilience against information loss (Voelkl and Noë, 2010). This study showed that information spreads faster in well-mixed groups compared to structured groups. In structured social networks, information also spreads faster when the frequency of interactions was either disregarded (unweighted or topological networks) or distributed randomly amongst interacting individuals. Similarly, the number of transmission events (path length) from an innovator individual to a target individual was greater in structured groups compared to well-mixed groups and was related to reduced connectivity and variation in interaction frequencies. Furthermore, variance in average path length was related to variation in group size, the larger the group the longer the path length, but also to community modularity, a measure that quantifies the structuring of a group into subgroups (Voelkl and Noë, 2010). Actually, there is more and more evidence that the structure of a social group, rather than its absolute size, influences network flow (e.g., pathogens or diseases: Griffin and Nunn, 2012; Nunn et al., 2015; emotions, tastes, or health outcomes: Fowler and Christakis, 2008; Bakshy et al., 2012; Christakis and Fowler, 2014). At a more global level, this is illustrated by the differences found in cooperative performance, enhanced in socially tolerant bonobos compared to more aggressive chimpanzees (Hare et al., 2007), or in socially tolerant Tonkean macaques compared to non-tolerant rhesus macaques (Petit et al., 1992). Those differences have been attributed to the fact that social networks of tolerant species are more diverse and open because individuals tolerate each other’s proximity better and this potentially offers a greater opportunity for information diffusion.

In humans, mathematical modeling has shown that social influences can lead to disproportionate diffusion of a trend or a fashion, an effect called the majority illusion (Lerman et al., 2015). In a network setting, behaviors can be contagious and spread to an entire population from a small subset of initial individuals. The speed and spread of this contagion has been shown to be heavily dependent on the network structure: a trend or a disease is transmitted faster if the initial adopters are very well connected and/or belong to very well connected clusters, e.g., superspreaders (Fujie and Odagaki, 2007; Garcia-Herranz et al., 2014). Because individuals take their social cues from their local neighbors, the characteristics and positions in the network of these initial adopters can greatly influence the contagion of a behavior, making it appear far more common locally than it is globally (Christakis and Fowler, 2014; Garcia-Herranz et al., 2014; Lerman et al., 2015). This has been termed the majority illusion and stems from the friendship paradox in which one’s friends appear to have more friends than one has (it also concerns tweets and academic citations for instance). The mathematical model developed by Lerman et al. (2015) quantifies the strength of this phenomenon and shows that it is stronger in networks with active high-degree nodes (active knowledgeable individuals) and heterogeneous degree distribution (because active knowledgeable individuals are more attractive and others in the population, non-active non-knowledgeable, pay more attention to them). Similarly, in health programs dedicated to educate people about hygiene and safer practices, targeting friends of individuals – themselves chosen randomly – in the population can have greater effects on the spread of behavioral changes than targeting individuals with the most social ties (Kim et al., 2015). This effect is attributed to the specific structuring of human social networks, which show subgroups of interconnected individuals each with their own locally influential nodes (Newman and Park, 2003; Fowler and Christakis, 2008; Kim et al., 2015). It also suggests that the assumption of greater centrality linked to greater influence on social processes is not always straightforward as this relationship can be mediated by sub-structuring, individual role or position, and synergies between indirect and direct connections. In fruit flies, social network structure [for example, homogeneous (individuals behave similarly) vs. heterogeneous] also affects information use, specifically in oviposition site choice: uninformed flies would either follow or avoid choices of informed flies depending on the amount of variance in individual network centrality among informed group mates, the greater the variance the more uninformed individuals avoided the same site as informed individuals (Pasquaretta et al., 2016). Social network modeling can thus improve the underpinning social variance and the understanding of why some behaviors spread – or on the contrary do not spread.

A factor that is often overlooked is that, although social life is extremely beneficial, it can also be stressful because individuals not only have to satisfy their own needs but also must do so while coordinating with the needs of others (Krause and Ruxton, 2002). Whether test subjects are in their social group settings or tested singly can have tremendous effects on their stress level and cause concomitant effects on decision-making in the laboratory or under natural conditions (van den Bos et al., 2013). As such, on the one hand experimental studies done in isolation of the social context may have little predictive value in terms of social information use in general, although they allow for the dissection of mechanisms and functions quite difficult to achieve in natural settings. On the other hand, the social group context can be very inhibiting for some individuals and thus can impede social information diffusion, such as potential or actual conflicts with conspecifics, or the fact that performing a task in front of conspecifics can be overwhelming (van den Bos et al., 2013). Stress affects memory and learning (Schwabe et al., 2012) and biases decisions (Aureli and Schino, 2004; Naqvi et al., 2006; Starcke and Brand, 2012). For example, individual ravens (*Corvus corax*) approach a novel object faster but spend less time interacting with it when alone than when in pairs or groups, seemingly trading off vigilance against innovation depending on risk and opportunity assessment (Stöwe et al., 2006). Brown rats (*Rattus norvegicus*) experiencing stress significantly and progressively lose the ability to adjust their responses toward a larger reward when transitioning from equal to unequal reward quantities (Graham et al., 2009). The effect of stressors on decision-making may not be of great consequence in animal social diffusion studies apart from failed experiments, but in humans, having to make a decision under high stress is linked to variation and volatility which likely reflects uncontrollability and unpredictability and can lead people or groups to make irrational choices (Starcke and Brand, 2012).

A final aspect of the influence of sociality on social information use is the social competence of animals. Social competence refers to the ability of individuals to regulate the expression of their social behavior in order to optimize their social relationships (Taborsky and Oliveira, 2012; Bshary and Oliveira, 2015). For instance, it allows individuals to avoid engaging in overly costly fights (“winner-loser” effect; Hsu et al., 2006; Taborsky and Oliveira, 2012) and to increase or decrease their degree of aggressiveness according to the familiarity of their opponents (familiar = “dear enemy” effect, stranger = “nasty neighbor” effect; Temeles, 1994; Taborsky and Oliveira, 2012). Social competence can also explain why individuals tend to cooperate more readily with social partners if they themselves have received help from others previously (“generalized reciprocity”; Pfeiffer et al., 2005; Taborsky and Oliveira, 2012). Although established from an evolutionary ecology point of view, with reference to phenotypic behavioral flexibility and plasticity, the vantage point of social competence provides an overview of the general ability and performance of individuals in a social environment (Taborsky and Oliveira, 2012). Recently, the social competence perspective has been paired with a game theoretic approach in animal cooperation with exactly this goal in mind. This more integrative framework also highlights the importance of studying the behavior and underlying decision rules/strategies of individuals across different social contexts, in the same way that behavioral syndromes encompass links and feedbacks of individual reaction norms across a variety of contexts (Bshary and Oliveira, 2015). Social diffusion studies would benefit enormously from taking such an integrative approach and accounting simultaneously for variation in the individual, social, and physical worlds.

## Smart Animals

Animals produce and receive, acquire and use social information from different individuals in different contexts and circumstances. The circumstances under which an animal uses social information rather than selects an option based on its own environmental sampling or the different rules animals adopt when making such decisions have been investigated in great details. Social diffusion experiments of all kinds are great tools to investigate the social insights of animals. Nevertheless, many important questions remain: how do animals distinguish informed and uninformed individuals? How do they judge the quality of a piece of information? What if several individuals are deemed knowledgeable but the information they provide conflict? What if the context in which social information is produced changes its value compared to another context? What if certain pieces of information are easier/less risky to get, but are also less accurate? To what extent the spread, reach and speed of transmission of a social information are affected by these parameters? Answering these questions, from our point of view, will require a more integrative approach, marrying different fields to reflect more realistically the probable holistic understanding animals have of their environments (Laland, 2004; Taborsky and Oliveira, 2012; Bshary and Oliveira, 2015).

On a practical side, with the accumulation of studies of diffusion, building a database of successful and failed experiments could better inform the scientific community. This could take the form of depositing protocols into an open-access database, such as the Dryad Digital Repository^1^, with the advantage of having corresponding digital object identifiers (doi), or creating a dedicated website on which to aggregate studies, pre-prints, and protocols in the same fashion as the Global Mammal Parasite Database^2^, with the advantage that it is searchable and collaborative. With the technology available today providing small cost-effective electronic devices [touch-screens, eye-trackers, automated feeders, accelerometers, radio-frequency identification (RFID) technology, GPS, etc.], broad-scale experiments and modeling could be possible as is now done regularly in cognitive science (Fagot and Bonté, 2010), ecology and social network studies (Rutz et al., 2012; Krause et al., 2013; Farine and Whitehead, 2015). One could setup providing automated food boxes with automatic food delivery devices and remote-controlled openings triggered by the approach of an animal equipped with RFID tags. Providing dozens of such boxes in a group setting would allow varying the quality, quantity, and reliability of the information available to group members both as producers and receivers. Tracking natural demographic changes or experimentally inducing changes by removing/adding individuals or manipulating the quality of a social bond could also give insights into the causes and consequences of social network structure on social information transmission.

This kind of diffusion experiments, with broad yet individualized parameters, could help tackle integrated questions related to variation and complexity of the environment, be it social or ecological. As has already been proposed for studies in cognitive science (see e.g., Barrett, 2011 and Wilson and Golonka, 2013 for an overview), social diffusion studies would also benefit from being more “embodied,” i.e., investigating social information use within individual, social and environmental contexts. Furthermore, studies on social information transmission could get inspiration from other domains such as epidemiology, informatics security, or social media, especially in humans, where studies also account for and integrate social network processes in empirical and mathematical studies, thereby providing tremendously important insights into biological and social processes. Finally, most of the experimental examples are situated in foraging, mating and anti-predator contexts, but far less has been done in social contexts such as aggression or affiliation. We know that animals are socially aware in the sense that they recognize their group mates or conspecifics, that they can keep track of their relationships and that they can use social concepts such as dominance and triadic relations (Whiten and Byrne, 1997; Dunbar, 1998; Emery, 2004; Holekamp et al., 2007; Silk, 2007). We have evidence that animals can recognize facial expression in conspecifics (Micheletta et al., 2015), that emotional arousal can spread through a group (collective arousal or emotion contagion, e.g., De Marco et al., 2011) and that animals can also judge and use the social reputation of others in their decisions (Alexander, 1987; Bshary and Bronstein, 2010). How animals make use of these kinds of social information to guide their decisions in their social relationships is an open field of investigation where social diffusion experiments can find their place. Better or further accounting for characteristics of information, of individuals, of cognitive and social competences is essential in making progress in the social information field and in the understanding of how animals make use – or not - of social information.

## Author Contributions

All authors made substantial contributions to the conception and design of the work; participated in the acquisition, analysis, or interpretation of data for the work; participated in drafting the work or revising it critically for important intellectual content; gave their final approval of the version to be published; and agree to be accountable for all aspects of the work.

## Conflict of Interest Statement

The authors declare that the research was conducted in the absence of any commercial or financial relationships that could be construed as a potential conflict of interest.

## Footnotes

1 http://datadryad.org/pages/organization

2 http://gmpd.nunn-lab.org/
